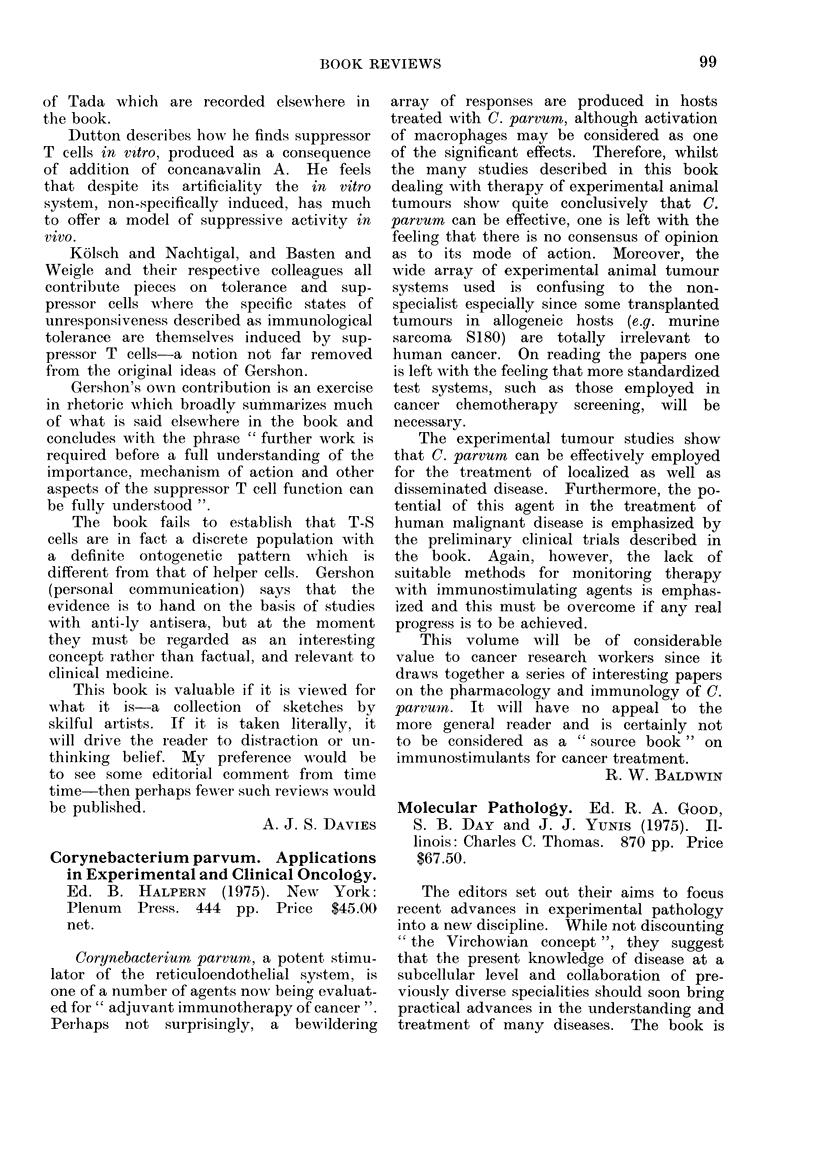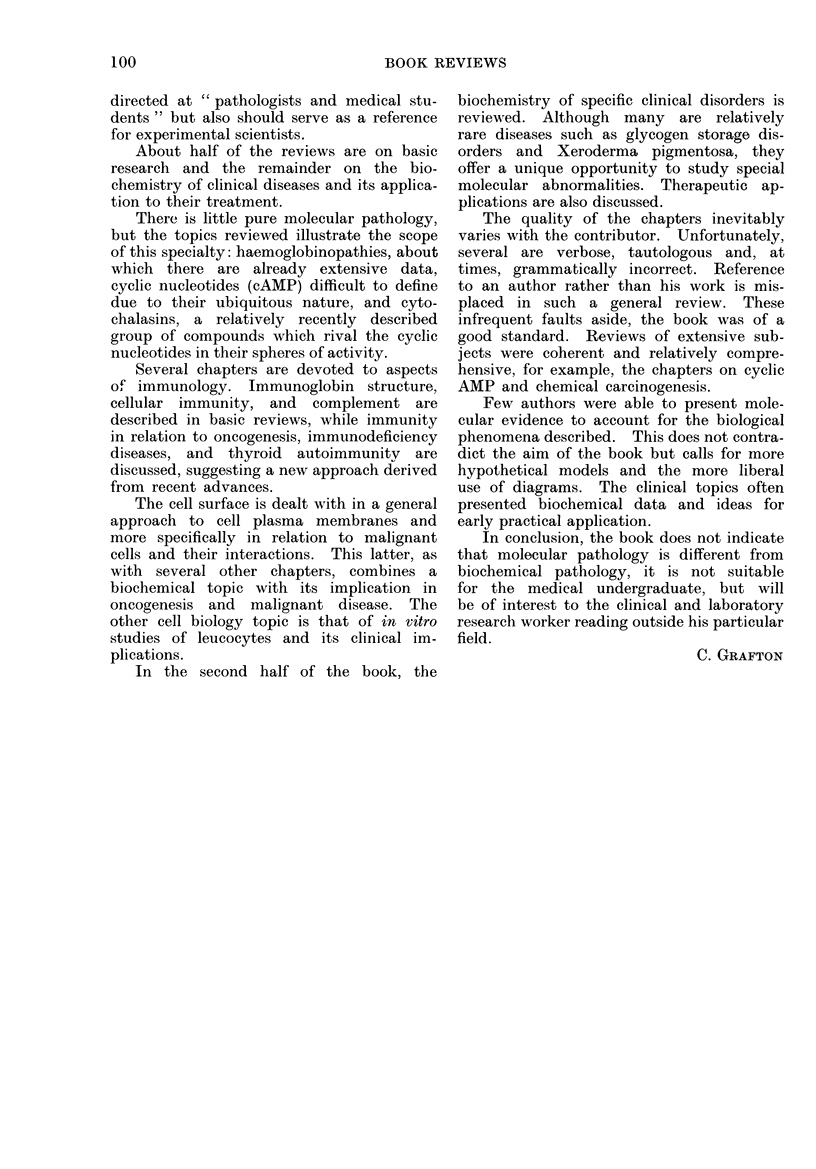# Molecular Pathology

**Published:** 1976-07

**Authors:** C. Grafton


**Molecular Pathology.** Ed. R. A. Good, S. B. Day and J. J. Yunis (1975). Illinois: Charles C. Thomas. 870 pp. Price $67.50.

The editors set out their aims to focus recent advances in experimental pathology into a new discipline. While not discounting " the Virchowian concept ", they suggest that the present knowledge of disease at a subcellular level and collaboration of previously diverse specialities should soon bring practical advances in the understanding and treatment of many diseases. The book is directed at " pathologists and medical students " but also should serve as a reference for experimental scientists.

About half of the reviews are on basic research and the remainder on the biochemistry of clinical diseases and its application to their treatment.

There is little pure molecular pathology, but the topics reviewed illustrate the scope of this specialty: haemoglobinopathies, about which there are already extensive data, cyclic nucleotides (cAMP) difficult to define due to their ubiquitous nature, and cytochalasins, a relatively recently described group of compounds which rival the cyclic nucleotides in their spheres of activity.

Several chapters are devoted to aspects of immunology. Immunoglobin structure, cellular immunity, and complement are described in basic reviews, while immunity in relation to oncogenesis, immunodeficiency diseases, and thyroid autoimmunity are discussed, suggesting a new approach derived from recent advances.

The cell surface is dealt with in a general approach to cell plasma membranes and more specifically in relation to malignant cells and their interactions. This latter, as with several other chapters, combines a biochemical topic with its implication in oncogenesis and malignant disease. The other cell biology topic is that of *in vitro* studies of leucocytes and its clinical implications.

In the second half of the book, the biochemistry of specific clinical disorders is reviewed. Although many are relatively rare diseases such as glycogen storage disorders and Xeroderma pigmentosa, they offer a unique opportunity to study special molecular abnormalities. Therapeutic applications are also discussed.

The quality of the chapters inevitably varies with the contributor. Unfortunately, several are verbose, tautologous and, at times, grammatically incorrect. Reference to an author rather than his work is misplaced in such a general review. These infrequent faults aside, the book was of a good standard. Reviews of extensive subjects were coherent and relatively comprehensive, for example, the chapters on cyclic AMP and chemical carcinogenesis.

Few authors were able to present molecular evidence to account for the biological phenomena described. This does not contradict the aim of the book but calls for more hypothetical models and the more liberal use of diagrams. The clinical topics often presented biochemical data and ideas for early practical application.

In conclusion, the book does not indicate that molecular pathology is different from biochemical pathology, it is not suitable for the medical undergraduate, but will be of interest to the clinical and laboratory research worker reading outside his particular field.

C. Grafton